# Disability-free life expectancy and life expectancy in good self-rated health in Chile: Gender differences and compression of morbidity between 2009 and 2016

**DOI:** 10.1371/journal.pone.0232445

**Published:** 2020-04-30

**Authors:** Ximena Moreno, Lydia Lera, Cecilia Albala

**Affiliations:** Institute of Nutrition and Food Technology, University of Chile, Santiago, Chile; Sciensano, BELGIUM

## Abstract

**Background:**

Chile has one of the highest life expectancies at 60 years in South America. This study was aimed to determine healthy life expectancies among Chilean older people, according to self-rated health and disability, and to explore gender differences.

**Methods:**

Data from the National Survey of Health (2009 and 2016) were used to estimate prevalence of less than good self-rated health and disability among people aged 60 years and above. Health expectancies were calculated with the Sullivan method.

**Results:**

In both years, women expected to live a lower proportion of their life expectancy in good self-rated health (54.5% [95% CI 50.0–58.8] for men and 37.6% [95% CI 34.3–40.8] for women in 2009; 46.1% [95% CI 42.6–49.7] for men and 38.5% [95% CI 35.6–41.4] for women in 2016). Life expectancy in less than good self-rated health increased for men (9.4 years [95% CI 8.4–10.3] in 2009; 11.5 years [95% CI 10.7–12.2]). Women expected to live a lower proportion of their remaining life without disabilities (65.3% [95% CI 61.2–69.4] for men and 44.9% [95% CI 41.9–47.9] for women in 2009; 71.9% [95% CI 68.7–75.0] for men and 61.1% [95% CI 58.5–63.8] for women in 2016). In 2016, disability-free life expectancy increased among women, but they still had a higher life expectancy with mild disability (2.8 years [95% CI 2.3–3.4] for men and 6.0 years [95% CI 5.4–6.7] for women).

**Conclusions:**

Women expected to spend more years in less than good self-rated health and disabled. There was an expansion of life expectancy in less than good SRH among men and a compression of disability in both sexes. The high proportion of years expected to be lived in less than good self-rated health and gender differences in disability-free life expectancy of older adults should be addressed by public health policies in Chile.

## Background

During the last century, a remarkable increase in life expectancy (LE) was observed, which resulted in a higher proportion of people reaching old age [1]. Aging of the population in Latin America has occurred at a higher speed, compared to many countries in Europe and North America, which had more time to adapt to changing social and economic demands [2]. In Chile, LE at birth in 2019 was 80 years [3]. Chile has one of the highest life expectancies at 60 years in South America. In 2017, women at 60 years had a LE of 24.6 years, and men of the same age expected to live 21.0 more years [4]. The global increase in LE has raised the question whether the additional years are lived in good health [5]. The theory of expansion of morbidity posits that an extension of LE has as a consequence the expansion of diseases and disabilities [6]. The compression of morbidity approach assumes that the burden of disease can be prevented and postponed and as LE extends, diseases and disabilities are reducing [7]. Compression or expansion of morbidity can be absolute or relative. Absolute expansion or compression of morbidity are expressed in more years lived with or without diseases or disabilities, respectively [8]. A decrease in the proportion of LE in good health combined with an increase in the number of years in good health is considered a relative expansion of morbidity, and a decrease of the proportion of LE in poor health, in combination with an increase in the number of years in poor health is considered a relative compression of morbidity [8]. Unlike the expansion of morbidity theory, the dynamic-equilibrium hypothesis states that severe morbidity does not increase as LE extends [9].

Health expectancies are measures that combine information on LE and health indicators, in order to estimate the remaining years to be lived in good health from a specific age [10]. Previous research on health expectancies has shown that there is an advantage for women in survival, but that they spend more years in worse health status, compared to men [11–13]. Specifically, older women are more likely to spend more years with negative self-rated health, functional impairment in instrumental activities of daily living, mobility limitations, depressive symptoms and cognitive impairment [14–18]. Several dimensions are included in possible explanations for the gender health-survival paradox. Biological differences could result in a higher resilience and better immune response among women [19,20]. Cultural patterns could also have an impact on lifestyles, with a higher tobacco and alcohol consumption [19], more frequent risk-taking behavior and a lower tendency to seek health care and to comply with treatments among men [20].

Health indicators to calculate health expectancies include self-rated health (SRH) and disability [21]. Although SRH is a subjective measure of health status, an important number of studies have shown its association with mortality among the older population [22–25], along with negative outcomes in terms of morbidity, falls and hospitalization [21]. According to results from the Chilean National Study of Dependency in Older People, 44.1% men and 54.2% women aged 60 years or more rated their health as less than good in 2009 [26]. Poor SRH among Chilean older women was associated with a higher risk of dying [27]. It has also been observed that Chilean older people with less chronic diseases were more likely to report good SRH [28]. Two Brazilian studies have reported LE in good SRH [29,30], but no information is available with respect to the Chilean population. Disability-free life expectancy (DFLE) has been used to monitor health expectancies of the population in the United States [31,32] and Europe [33–35] over the past several decades. Several studies on DFLE including Latin American populations exist [36–45], but only two studies have reported DFLE among Chilean older people, and they have focused on the population living in Santiago [46,47]. Estimations of LE in good SRH and DFLE at national level and comparisons over time are needed.

This study was aimed to estimate LE in good SRH and DFLE among Chilean men and women aged 60 years in 2009 and 2016, and to explore gender differences.

## Methods

### Data

We analyzed data from the National Survey of Health (NSH), a Chilean cross-sectional study with three waves. Data from the two last waves (2009 and 2016) were employed. Stratified cluster sampling was used in both waves, based on the master sample frame of the Chilean National Institute of Statistics and the Population and Housing Census of Chile [48,49]. The samples for the two versions included people aged 15 years or more (n = 5293 in 2009; n = 6233 in 2016) and were nationally representative. Taking into account the complex study designs, weights for both datasets were calculated [48,50].

The protocols of the NSH 2009 and 2016 were approved by the Ethics Committee of the Pontificia Universidad Católica de Chile (Pontifical Catholic University of Chile). Participants signed informed consent to take part in the NSH. The databases were fully anonymized by the Chilean Ministry of Health, before they were made publicly available for research purposes.

Data collection was carried out via face to face household interviews. A detailed set of sociodemographic and health variables were gathered. The question employed to assess SRH was: “In general, would you say your health is?” The possible answers were: Very good, good, fair, poor, very poor. The answers were collapsed into good (very good and good) and less than good (fair, poor and very poor). Disability was determined according to the definition proposed by Albala et al. [51] and Fuentes-Garcia et al. [52], based on information about activities of daily living (ADL), instrumental activities of daily living (IADL) and cognitive status, ascertained by a modified version of the Mini-Mental State Examination (MMSE) and the Pfeffer Functional Activities Questionnaire (PFAQ), which have been validated as a screening test for cognitive impairment among the Chilean population [53,54]. In 2009, ADLs included were: bathing, dressing and grooming. IADLs included: housekeeping activities and transportation. In 2016, ADLs were: dressing, eating, toileting and transferring from a bed. IADLs were: housekeeping activities, transportation and shopping. Participants were classified as without disability or with disability (mild or severe), according to the following criteria:

- Without disability: no difficulties or difficulties in one IADL. No difficulties in ADL.
- Mild disability: difficulties in two IADL or MMSE<13 and PFAQ>5 and <12. No difficulties in ADL.
- Severe disability: difficulties in one or more ADLs or MMSE<13 and PFAQ>11.

### Health expectancies calculation

Abridged life tables for the Chilean population in 2009 and 2016 were used. Life tables were retrieved from the Global Health Observatory Data Repository of the World Health Organization. Weighted sex-specific prevalence of less than good SRH and disability was estimated for each five year age group. The Sullivan method [55] was employed to calculate years of life expected to be lived in good SRH and free of disability, based on LE at specific ages and estimated prevalence of less than good SRH and disability. Based on the proposal by Jagger et al. [56], we followed these steps: 1. Calculation of person years to be lived in good SRH/disability-free, by multiplying the person years lived in a specific age group by the proportion of people with good SRH/disability-free (1-weighted prevalence). 2. Addition of the person years with good SRH/disability-free of the successive age intervals, to calculate the total number of years in good SRH/disability-free. 3. Calculation of LE in good SRH/disability-free, by dividing the total years lived in good SRH/disability-free by the number surviving to each age interval. Standard errors were calculated to obtain 95% confidence intervals. This process considered: 1.Calculation of the variance of the prevalence (product of the prevalence by its complement, divided by the number of participants in an age interval). 2. Sum of the product of the square of the person years lived in an age interval and the variance of the prevalence rate, from a specific age interval to the final age interval. 3. To obtain the variance, the previous result for each age interval was divided by the square of the number of people surviving to the beginning of the age interval. 4. The standard error was the square root of the variance of the health expectancy.

Stata 15 was employed for descriptive statistics and prevalence estimation. Life expectancies and health expectancies were calculated with R 3.6.1.

## Results

The NSH 2009 included a total of 1390 participants aged 60 years or more, and the NSH 2016 had a sample of 2031 people of the same age (Table 1).

**Table 1:** Sample size by age group in the National Survey of Health, Chile, 2009 and 2016.

	2009		2016	
	Men	Women	Men	Women
	(n = 543)	(n = 847)	(n = 737)	(n = 1294)
Age groups (%)				
60–64	28.2	26.5	26.7	24.5
65–69	23.8	23.3	24.2	23.7
70–74	19.2	16.1	19.5	19.5
75–79	16.2	16.1	13.0	15.5
80–84	8.7	11.1	10.2	10.4
85+	4.1	7.1	6.4	6.6

In 2016, a higher proportion of women had no education or had completed less than primary level of school (less than 8 years) and a higher proportion of men had attended at least one year of higher education (13 or more years) (Table 2). More than four fifths of men and women lived in urban areas. A lower proportion of women had private health insurance in 2016.

**Table 2:** Sociodemographic characteristics: National Survey of Health, Chile, 2009 and 2016 (weighted percentages).

	2009			2016		
	Men	Women	P value	Men	Women	P value
Years of education (%)			.216			.002
**0**	6.0	6.0		3.4	5.7	
1–7	39.9	49.3		37.5	45.1	
8–12	38.2	33.4		37.6	38.5	
13+	15.8	11.3		21.5	10.7	
Area of residence (%)			.943			.197
Urban	83.6	83.4		84.2	86.9	
Rural	16.4	16.6		15.8	13.1	
Health insurance (%)			.795			.003
Public	83.8	84.7		82.6	91.3	
Private	16.2	15.3		17.4	8.7	

As observed in Table 3, prevalence of less than good SRH was higher among women. Mild disability decreased across time among women, but the prevalence was higher compared to men in both years.

**Table 3:** Prevalence of less than good self-rated health and disability among Chilean older men and women, 2009 and 2016.

	2009		2016	
	%	95% CI	%	95% CI
Less than good SRH				
Total	52.3	47.6–57.0	56.0	52.0–60.0
Men	44.3	36.6–52.1	50.0	43.6–56.5
Women	58.9	53.4–64.3	61.4	56.7–66.2
Any level of disability				
Total	40.0	35.5–44.4	27.6	24.2–31.1
Men	30.5	23.6–37.4	23.8	18.2–29.4
Women	47.7	42.1–53.3	31.1	26.8–35.3
Mild disability				
Total	20.1	16.5–23.7	14.4	11.8–17.1
Men	14.1	9.0–19.2	10.0	6.1–13.8
Women	25.0	20.1–30.0	18.5	14.9–22.1
Severe disability				
Total	20.0	16.5–23.2	13.2	10.5–15.8
Men	16.4	11.0–21.8	13.9	9.3–18.5
Women	22.7	18.4–27.0	12.5	9.7–15.4

In 2016, LE at age 60 was 21.3 years for men and 25.3 years for women, showing an increase of 0.8 years among men and 0.4 years among women. Sex differences in LE in good SRH were observed (Table 4). Men and women expected to live a similar number of years in good SRH, but women expected to live more years with less than good SRH (6 more years in 2009; 4.1 more years in 2016) and had a higher proportion of years to be lived in less than good SRH in 2009 (16.2 percent points) and 2016 (7.6 percent points), compared to men. LE in less than good SRH increased 2.1 years for men across time.

**Table 4:** Total life expectancy, life expectancy in good self-rated health and life expectancy in less than good self-rated health at age 60 in Chile, 2009 and 2016.

	2009		2016	
	Men	Women	Men	Women
Life expectancy (years)	20.5	24.9	21.3	25.3
HLE (95% CI)	11.1 (10.2–12.1)	9.5 (8.7–10.3)	9.8 (9.1–10.6)	9.7 (9.0–10.5)
% HLE (95% CI)	54.5 (50.0–58.8)	38.2 (34.9–41.5)	46.1 (42.6–49.7)	38.5 (35.6–41.4)
ULE (95% CI)	9.4 (8.4–10.3)	15.4 (14.6–16.2)	11.5 (10.7–12.2)	15.6 (14.8–16.3)
% ULE (95% CI)	45.6 (41.2–50.0)	61.8 (58.5–65.1)	53.9 (50.3–57.4)	61.5 (58.6–64.4)

HLE = life expectancy in good SRH, ULE = life expectancy in less than good SRH.

**Fig 1:**
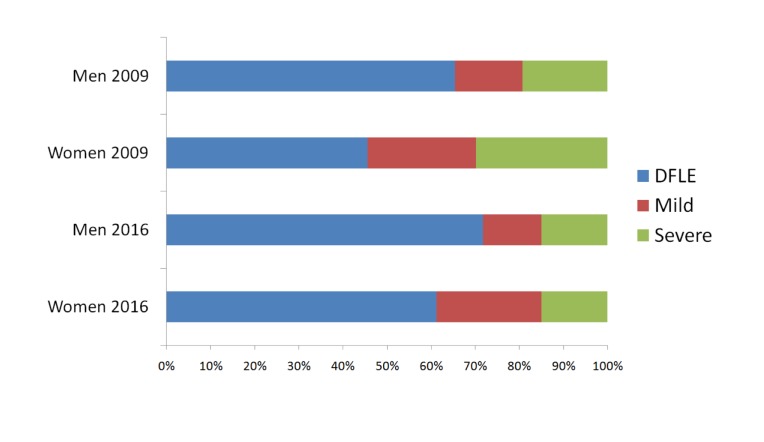
Proportion of disability-free life expectancy and of life expectancy with mild and severe disability among Chilean older men and women, 2009 and 2016.

As shown in Table 5, women had a higher disabled life expectancy (DLE) (6.4 more years in 2009; 3.8 more years in 2016), compared to men. Fig 1 shows that the proportion of years to be lived with disabilities was higher for women (19.7 more percent points in 2009 and 10.8 more percent points in 2016). In 2016, disability-free life expectancy increased among men and women, but women still had a higher life expectancy with mild disability, compared to men.

**Table 5:** Total life expectancy, disability-free life expectancy and disabled life expectancy at age 60 in Chile, 2009 and 2016.

	2009		2016	
	Men	Women	Men	Women
Life expectancy (years)	20.5	24.9	21.3	25.3
DFLE (95% CI)	13.4 (12.5–14.2)	11.4 (10.6–12.1)	15.3 (14.6–16.0)	15.5 (14.8–16.1)
% DFLE (95% CI)	65.3 (61.2–69.4)	45.6 (42.6–48.7)	71.9 (68.7–75.0)	61.1 (58.5–63.8)
DLE (95% CI)	7.1 (6.3–8.0)	13.5 (12.8–14.3)	6.0 (5.3–6.7)	9.8 (9.2–10.5)
% DLE (95% CI)	34.7 (30.6–38.8)	54.4 (51.3–57.4)	28.1 (25.0–31.2)	38.9 (36.2–41.5)
MDLE (95% CI)	3.1 (2.5–3.7)	6.1 (5.4–6.9)	2.8 (2.3–3.4)	6.0 (5.4–6.7)
% MDLE (95% CI)	15.3 (12.3–18.3)	24.6 (21.5–6.9)	13.3 (10.9–15.8)	23.8 (21.2–26.4)
SDLE (95% CI)	3.9 (3.3–4.7)	7.4 (6.7–8.2)	3.2 (2.7–3.8)	3.8 (3.2–4.3)
% SDLE (95% CI)	19.3 (15.7–22.8)	29.9 (26.9–32.9)	15.1 (12.5–17.6)	14.9 (12.6–17.1)

DLE = life expectancy with any disability, MDLE = life expectancy with mild disability, SDLE = life expectancy with severe disability.

## Discussion

According to our results, Chilean women aged 60 years expected to live a higher proportion of years in less than good SRH in 2009 and 2016, compared to men. The proportion of DLE was higher among women. In particular, they expected to live a higher proportion of their lives with mild disability, compared to men. There was an absolute expansion of LE in less than good SRH for men, and an absolute compression of disability for both sexes.

A recent study by our group analyzed data from the NSH from 2003 to 2016, to estimate life expectancy free of cognitive impairment [57]. That report found no sex difference in the proportion of years to be lived free of cognitive impairment between Chilean men and women aged 60 years. A higher LE among women resulted in more years to be lived free of CI, compared to men. Hence, gender differences in health expectancies among the Chilean older population vary depending on the health indicator used.

Sex differences with respect to LE in less than good SRH have been previously reported. Unlike our results, other studies in England [58] and Brazil [29,30] found a higher LE in good SRH among older women. In England, older women also had a longer LE in less than good SRH, compared to men [59].

Brazilian studies that focused on LE in good SRH [29,30] reported considerably higher LE in good SRH for Brazilian men and women aged 60, compared to our results. LE in good SRH at 60 years was 16.8 years for Brazilian men and 20.8 years for Brazilian women in 2008 [29]. In 2013, LE in good SRH at the same age was 17.3 years for Brazilian men and 20.2 years for Brazilian women [30]. According to Jagger et al. [58], English older people in 2011 had higher LE in good SRH at 65 years (12.6 years for men; 14.3 years for women), compared to Chilean older men and women aged 60 in 2009 and 2016. Chilean men and women expected to live more than half of their LE at 60 years in less than good SRH, which is much higher than what other studies have reported [29,30,58].

Although SRH is a subjective measure, a substantial number of studies have found that a negative SRH is associated with mortality among older adults [22–25]. Accordingly, a study found that poor SRH increased the risk of mortality among Chilean older women [27]. Some studies have shown that the validity of SRH as a predictor of mortality varies depending on educational attainment [59,60]. Literacy among the population 15 years or older in Chile is 95.7% [61]. The NSH did not measure literacy, but no education can be considered a proxy for illiteracy, since during the first year of schooling reading and writing are taught in Chile. We observed a slightly higher proportion of people with no education in 2009, which persisted in 2016 among women (Table 1). A previous study from 2009 reported a higher level of illiteracy among rural, population aged 60 years or more in Chile, compared to urban population of the same age [26]. The proportion of older people living in rural areas in Chile in 2016 was 15.8% for men and 13.1% for women. The accuracy of SRH as a health status indicator among Chilean rural older population and those groups with lower education should be further examined.

Our findings showed a high proportion of years expected to be lived in less than good SRH among Chilean older people, which did not decrease between 2009 and 2016. Therefore, more research is needed to disentangle the factors associated with the high prevalence of less than good SRH among the Chilean older population. Chilean public policies should address the high proportion of LE in less than good SRH observed among older men and women.

Chilean older women expected to live a lower proportion of their lives without disability and a higher proportion with disabilities, compared to men. Similar findings have been reported in other countries by previous studies [38,40,58]. In 2009, LE with mild or severe disability was higher for women. A previous longitudinal study found that up to 2010 women aged 60 years expected to live a higher proportion of their lives with any level of disability, compared to men of the same age in Santiago, Chile [47]. Data from Brazil in 2008 [29] and England in 2011 [58] showed the same sex difference. However, in 2016 only LE with moderate disability was higher for Chilean women.

An analysis of trends in DFLE in Brazil between 2000 and 2010 found an expansion of severe disability [40]. For the period 1991–2011 in England, Jagger et al. [58] also reported an increase of DLE, with an expansion of the proportion of life with mild disability only. According to our results in Chile, between 2009 and 2016, LE with any level of disability remained stable for men and LE with severe disability decreased for women.

This is the first study to report LE in good SRH and DFLE among Chilean older people at a national level. To date, there were no analyses of LE in good SRH or trends in DFLE in Chile. It is important to consider some limitations. Only cross-sectional data were available to make comparisons between different points in time. However, this approach is widely used in health expectancies studies and it was used in most of the studies discussed above [29,30,58]. Second, European studies have shown that people with less education, lower socioeconomic position or with poor health have a lower participation rate in surveys [62,63]. If this is the case in the NSH, the health status of the Chilean older population could be overestimated. Thirdly, there were differences in the number of items measuring ADL and IADL in the NSH between 2009 and 2016, with two more items considered the last year. A lower number of questions about these difficulties could have decreased the probability of reporting IADL or ADL limitations in 2009. The observed decrease in disability prevalence could have been underestimated. Lastly, previous research has found social inequalities in health status and disability trajectories in Chile [52,64]. Furthermore, a recent study reported differences of 8.9 years for men and 17.7 years for women in LE at birth, between the most deprived and the least deprived areas of Santiago, the capital city of Chile [65]. The data considered in this study did not allow to calculate life tables by socioeconomic position or years of education. Hence, it was not possible to examine the impact of socioeconomic factors in health expectancies.

## Conclusions

This is the first study to analyze LE in good SRH among Chilean older adults. Also, this is the first report that compares DFLE at different points in time in Chile. Longer female LE was accompanied by more years in less than good self-rated health and disability for women. During the period considered, less than good SRH expanded among men. There has been a compression of severe disability among women, but mild disability has remained stable between 2009 and 2016. The high proportion of years expected to be lived in less than good self-rated health and gender differences in disability-free life expectancies of older adults should be addressed by public health policies in Chile.

## References

[1] Crimmins EM. Lifespan and Healthspan: Past, Present, and Promise. Gerontologist. 2015;55(6):901–911. 10.1093/geront/gnv130 26561272 PMC4861644

[2] Palloni A, McEniry M. Aging and health status of elderly in Latin America and the Caribbean: preliminary findings. J Cross Cult Gerontol. 2007 9;22(3):263–85. 10.1007/s10823-006-9001-7 17021957

[3] United Nations, Department of Economic and Social Affairs, Population Division (2019). World Population Prospects 2019: Data Booklet (ST/ESA/SER.A/424).

[4] United Nations, Department of Economic and Social Affairs, Population Division (2017). World Population Ageing 2017—Highlights (ST/ESA/SER.A/397).

[5] Beltrán-Sánchez H, Soneji S, Crimmins EM. Past, Present, and Future of Healthy Life Expectancy. Cold Spring Harb Perspect Med. 2015;5(11):a025957 10.1101/cshperspect.a025957 26525456 PMC4632858

[6] Kramer M 1980: Kramer M. The rising pandemic of mental disorders and associated chronic diseases and disabilities. Acta Psychiatr Scand. 1980;62((S285)):382–97.7468297

[7] Fries et al, 1989: Fries J, Green L, Levine S. Health promotion and the compression of morbidity. The Lancet. 1989;333((8636)):481–3. 10.1016/s0140-6736(89)91376-7 2563849

[8] Nussleder2003: Nusselder WJ. Compression of morbidity. In: Robine J-M, Jagger C, Mathers CD, Crimmins EM, Suzman RM, editors. Determining health expectancies John Wiley and Sons; Chichester, UK: 2003 pp. 35–58.

[9] Manton KG. Changing concepts of morbidity and mortality in the elderly population. Milbank Mem Fund Q Health Soc. 1982;60:183–244 6919770

[10] Stifel et al 2010: Stiefel MC, Perla RJ, Zell BL. A healthy bottom line: healthy life expectancy as an outcome measure for health improvement efforts. Milbank Q. 2010;88(1):30–53. 10.1111/j.1468-0009.2010.00588.x 20377757 PMC2888015

[11] Cheung KS, Yip PS. Trends in healthy life expectancy in Hong Kong SAR 1996–2008. Eur J Ageing. 2010;7(4):257–269. 10.1007/s10433-010-0171-3 21212818 PMC2995861

[12] Van Oyen H, Nusselder W, Jagger C, Kolip P, Cambois E, Robine JM. Gender differences in healthy life years within the EU: an exploration of the "health-survival" paradox. Int J Public Health. 2013;58(1):143–155. 10.1007/s00038-012-0361-1 22618297 PMC3557379

[13] Nusselder et al 2019: Nusselder WJ, Cambois EM, Wapperom D, et al Women's excess unhealthy life years: disentangling the unhealthy life years gap. Eur J Public Health. 2019;29(5):914–919. 10.1093/eurpub/ckz114 31280299 PMC6761840

[14] Zunzunegui MV, Alvarado BE, Béland F, Vissandjee B. Explaining health differences between men and women in later life: a cross-city comparison in Latin America and the Caribbean. Soc Sci Med. 2009 1;68(2):235–42. 10.1016/j.socscimed.2008.10.031 19036488

[15] Crimmins EM, Kim JK, Solé-Auró A. Gender differences in health: results from SHARE, ELSA and HRS. Eur J Public Health. 2011;21(1):81–91. 10.1093/eurpub/ckq022 20237171 PMC3023013

[16] Oksuzyan A, Crimmins E, Saito Y, O'Rand A, Vaupel JW, Christensen K. Cross-national comparison of sex differences in health and mortality in Denmark, Japan and the US. Eur J Epidemiol. 2010;25(7):471–480. 10.1007/s10654-010-9460-6 20495953 PMC2903692

[17] Trujillo AJ, Mroz TA, Piras C, Vernon JA, Angeles G. Determinants of gender differences in health among the elderly in Latin America. World Health Popul. 2010;11(3):24–43. 10.12927/whp.2010.21663 20357557

[18] Oksuzyan A, Shkolnikova M, Vaupel JW, Christensen K, Shkolnikov VM. Sex differences in health and mortality in Moscow and Denmark. Eur J Epidemiol. 2014;29(4):243–252. 10.1007/s10654-014-9893-4 24668060 PMC4090601

[19] Carmel S. Health and Well-Being in Late Life: Gender Differences Worldwide. Front Med (Lausanne). 2019;6:218 Published 2019 Oct 10. 10.3389/fmed.2019.00218 31649931 PMC6795677

[20] Oksuzyan A, Juel K, Vaupel JW, Christensen K. Men: good health and high mortality. Sex differences in health and aging. Aging Clin Exp Res. 2008;20(2):91–102. 10.1007/bf03324754 18431075 PMC3629373

[21] Saito et al 2018: Saito Y, Robine JM, Crimmins EM. The methods and materials of health expectancy. Stat J IAOS. 2014;30(3):209–223. 10.3233/SJI-140840 30319718 PMC6178833

[22] Idler EL and Benyamini Y. 1997. Self-rated health and mortality: A review of 27 community studies. J Health Soc Behav. 1997;38:21–37. 9097506

[23] Benyamini Y and Idler EL. 1999 Community studies reporting association between self-rated health and mortality: Additional studies, 1995–1998. Res Aging, 21: 392–401.

[24] DeSalvo KB, Bloser N, Reynolds K, He J, Muntner P. Mortality prediction with a single general self-rated health question. A meta-analysis. J Gen Intern Med. 2006;21:267–75. 10.1111/j.1525-1497.2005.00291.x 16336622 PMC1828094

[25] Moreno X, Huerta M, Albala C. Autopercepción de salud general y mortalidad en adultos mayores [Global self-rated health and mortality in older people]. Gac Sanit. 2014; 10.1016/j.gaceta.2013.07.006 24359681

[26] Albala C, Sanchez H, Fuentes A, Lera L, Cea X, Salas F et al. (2010) Estudio Nacional de la Dependencia en las Personas Mayores (National Survey of Dependence among Older Adults). http://www.senama.cl/filesapp/Estudio_dependencia.pdf. Accessed 14 Oct 2019.

[27] Moreno X, Albala C, Lera L, Sánchez H, Fuentes-García A, Dangour AD. The role of gender in the association between self-rated health and mortality among older adults in Santiago, Chile: A cohort study. PLoS One. 2017 7 18;12(7):e0181317 10.1371/journal.pone.0181317 28719627 PMC5515418

[28] Campos AC, Albala C, Lera L, Sánchez H, Vargas AM, Ferreira e Ferreira E. Gender differences in predictors of self-rated health among older adults in Brazil and Chile. BMC Public Health. 2015 4 11;15:365 10.1186/s12889-015-1666-9 25884800 PMC4432978

[29] Belon AP, Lima MG, Barros MB. Gender differences in healthy life expectancy among Brazilian elderly. Health Qual Life Outcomes. 2014;12:88 10.1186/1477-7525-12-88 24906547 PMC4079932

[30] Szwarcwald CL, Souza Júnior PR, Marques AP, Almeida WD, Montilla DE. Inequalities in healthy life expectancy by Brazilian geographic regions: findings from the National Health Survey, 2013. Int J Equity Health. 2016; 10.1186/s12939-016-0432-7 27852270 PMC5112675

[31] Crimmins EM, Zhang Y, Saito Y. Trends Over 4 Decades in Disability-Free Life Expectancy in the United States. Am J Public Health. 2016;106(7):1287–1293. 10.2105/AJPH.2016.303120 27077352 PMC4984740

[32] Freedman VA, Wolf DA, Spillman BC. Disability-Free Life Expectancy Over 30 Years: A Growing Female Disadvantage in the US Population. Am J Public Health. 2016;106(6):1079–1085. 10.2105/AJPH.2016.303089 26985619 PMC4860065

[33] Perenboom RJ, Van Herten LM, Boshuizen HC, Van Den Bos GA. Trends in disability-free life expectancy. Disabil Rehabil. 2004 4 8;26(7):377–86. 10.1080/0963828032000174098 15204474

[34] Frova L, Burgio A, Battisti A. Are gaps in disability free life expectancies diminishing in Italy?. Eur J Ageing. 2010;7(4):239–247. 10.1007/s10433-010-0173-1 28798632 PMC5547328

[35] Cambois E, Blachier A, Robine JM. Aging and health in France: an unexpected expansion of disability in mid-adulthood over recent years. Eur J Public Health. 2013 8;23(4):575–81. 10.1093/eurpub/cks136 23042230

[36] Camargos MC, Machado CJ, do Nascimento Rodrigues R. Disability life expectancy for the elderly, city of Sao Paulo, Brazil, 2000: gender and educational differences. J Biosoc Sci. 2007 5;39(3):455–63. 10.1017/S0021932006001428 16707040

[37] Rose AM, Hennis AJ, Hambleton IR. Sex and the city: differences in disease- and disability-free life years, and active community participation of elderly men and women in 7 cities in Latin America and the Caribbean. BMC Public Health. 2008 4 21;8:127 10.1186/1471-2458-8-127 18426599 PMC2387143

[38] Drumond Andrade FC, Guevara PE, Lebrão ML, de Oliveira Duarte YA, Santos JL. Gender differences in life expectancy and disability-free life expectancy among older adults in São Paulo, Brazil. Womens Health Issues. 2011 Jan-Feb;21(1):64–70. 10.1016/j.whi.2010.08.007 21185991

[39] Beltrán-Sánchez H, Andrade FC. Educational and sex differentials in life expectancies and disability-free life expectancies in São Paulo, Brazil, and urban areas in Mexico. J Aging Health. 2013;25(5):815–838. 10.1177/0898264313491425 23781016 PMC3845480

[40] Campolina AG, Adami F, Santos JLF, Lebrao ML. Expansion of morbidity: trends in healthy life expectancy of the elderly population. Rev. Assoc. Med. Bras. 2014;60(5):434–441.

[41] Santosa A, Schröders J, Vaezghasemi M, Ng N. Inequality in disability-free life expectancies among older men and women in six countries with developing economies. J Epidemiol Community Health. 2016;70(9):855–861. 10.1136/jech-2015-206640 26994068 PMC5013163

[42] Chirinda W, Chen H. Comparative study of disability-free life expectancy across six low- and middle-income countries. Geriatr Gerontol Int. 2017 4;17(4):637–644. 10.1111/ggi.12748 27197085

[43] Payne CF. Aging in the Americas: Disability-free Life Expectancy Among Adults Aged 65 and Older in the United States, Costa Rica, Mexico, and Puerto Rico. J Gerontol B Psychol Sci Soc Sci. 2018;73(2):337–348. 10.1093/geronb/gbv076 26347520 PMC6283317

[44] Camargos MCS, Gonzaga MR, Costa JV, Bomfim WC. Disability-free life expectancy estimates for Brazil and Major Regions, 1998 and 2013. Cien Saude Colet. 2019 3;24(3):737–747. 10.1590/1413-81232018243.07612017 30892496

[45] Prina AM, Wu YT, Kralj C, Acosta D, Acosta I, Guerra M, et al Dependence- and Disability-Free Life Expectancy Across Eight Low- and Middle-Income Countries: A 10/66 Study. J Aging Health. 2019 1 30:898264319825767 10.1177/0898264319825767 30698491 PMC7322974

[46] Minicuci N, Noale M, León Díaz EM, Gómez León M, Andreotti A, Mutafova M. Disability-free life expectancy: a cross-national comparison among Bulgarian, Italian, and Latin American older population. J Aging Health. 2011 6;23(4):629–81. 10.1177/0898264310390940 21220352

[47] Moreno X, Albala C, Lera L, Leyton B, Angel B, Sánchez H. Gender, nutritional status and disability-free life expectancy among older people in Santiago, Chile. PLoS One. 2018; 10.1371/journal.pone.0194074 29590148 PMC5874002

[48] Ministry of Health. Encuesta Nacional de Salud ENS Chile 2009–2010 [National Survey of Health ENS Chile 2009–2010]. https://www.minsal.cl/portal/url/item/bcb03d7bc28b64dfe040010165012d23.pdf Accessed 4 July 2019.

[49] Ministry of Health. Encuesta Nacional de Salud 2016–2017. Diseño Muestral [National Survey of Health 2016–17. Sampling design]. http://epi.minsal.cl/wp-content/uploads/2018/05/DISE%C3%91O-MUESTRAL-ENS-2016-2017.pdf Accessed 5 July 2019.

[50] Ministry of Health. Encuesta Nacional de Salud 2016–2017. Cálculo de Factores de Expansión [National Survey of Health 2016–17. Calculation of Sample Weights]. http://epi.minsal.cl/wp-content/uploads/2018/06/Informe-c%C3%A1lculo-de-factores-de-expansi%C3%B3n-ENS-2016-2017.pdf Accessed 8 July 2019.

[51] Albala C, Lera L, García C, Arroyo P, Marín PP, Bunout D. Searching a Common Definition for Functional Limitation in Latin America. Gerontologist. 2004;44: 550s.

[52] Fuentes-García A, Sánchez H, Lera L, Cea X, Albala C. Desigualdades socioeconómicas en el proceso de discapacidad en una cohorte de adultos mayores de Santiago de Chile (Socioeconomic inequalities in the onset and progression of disability in a cohort of older people in Santiago, Chile).Gac Sanit. 2013;27: 226–232. 10.1016/j.gaceta.2012.11.005 23291031

[53] Icaza MG, Albala C. Proyecto SABE. Minimental State Examinations (MMSE) del estudio de demencia en Chile: Análisis estadístico [SABE Project. Minimental State Examinations (MMSE) from the Study of Dementia in Chile: Statistical Analysis]. Washington, D.C.; Panamerican Health Organization 4, 1999.

[54] Quiroga P, Albala C, Klaasen G. Validación de un test de tamizaje para el diagnóstico de demencia asociada a edad, en Chile [Validation of a screening test for age associated cognitive impairment, in Chile]. Rev Med Chil. 2004;132:467–78. 10.4067/s0034-98872004000400009 15382519

[55] Sullivan DF. A single index for mortality and morbidity. HSMHA Health Rep. 1971;86:347–54. 5554262 PMC1937122

[56] Jagger C, Van Oyen H, Robine JM. Health Expectancy Calculation by the Sullivan Method: A Practical Guide. 4th ed. European Health Expectancy Monitoring Unit (EHEMU), 2014.

[57] Moreno X, Lera L, Moreno F, Albala C. Life expectancy with and without cognitive impairment among Chilean older adults: results of the National Survey of Health (2003, 2009 and 2016). BMC Geriatr. 2019;19(1):374 Published 2019 Dec 26. 10.1186/s12877-019-1387-5 31878877 PMC6933700

[58] Jagger C, Matthews FE, Wohland P, Fouweather T, Stephan BC, Robinson L, et al A comparison of health expectancies over two decades in England: results of the Cognitive Function and Ageing Study I and II. Lancet. 2016; 10.1016/S0140-6736(15)00947-2 26680218 PMC4761658

[59] Dowd JB, Zajacova A. Does the predictive power of self-rated health for subsequent mortality risk vary by socioeconomic status in the US? Int J Epidemiol. 2007 12;36(6):1214–21. 10.1093/ije/dym214 17971388

[60] Regidor E, Guallar-Castillón P, Gutiérrez-Fisac JL, Banegas JR, Rodríguez-Artalejo F. Socioeconomic variation in the magnitude of the association between self-rated health and mortality. Ann Epidemiol. 2010 5;20(5):395–400. 10.1016/j.annepidem.2010.01.007 20382341

[61] Gitlin LN, Fuentes P. The Republic of Chile: an upper middle-income country at the crossroads of economic development and aging. Gerontologist. 2012;52(3):297–305. 10.1093/geront/gns054 22534464 PMC4047290

[62] Lorant V, Demarest S, Miermans PJ, Van Oyen H. Survey error in measuring socio-economic risk factors of health status: a comparison of a survey and a census. Int J Epidemiol. 2007 12;36(6):1292–9. 10.1093/ije/dym191 17898025

[63] Spitzer S. Biases in health expectancies due to educational differences in survey participation of older Europeans: It’s worth weighting for. Eur J Health Econ. 2020 10.1007/s10198-019-01152-0 PMC7214500 31989388

[64] Albala C, Sánchez H, Lera L, Angel B, Cea X. Efecto sobre la salud de las desigualdades socioeconómicas en el adulto mayor. Resultados basales del estudio expectativa de vida saludable y discapacidad relacionada con la obesidad (Alexandros) [Socioeconomic inequalities in active life expectancy and disability related to obesity among older people]. Rev Med Chil. 2011; /S0034-98872011001000005. 22286726

[65] Bilal U, Alazraqui M, Caiaffa WT, Lopez-Olmedo N, Martinez-Folgar K, Miranda JJ, et al Inequalities in life expectancy in six large Latin American cities from the SALURBAL study: an ecological analysis [published correction appears in Lancet Planet Health. 2020 Jan;4(1):e11]. Lancet Planet Health. 2019;3(12):e503–e510. 10.1016/S2542-5196(19)30235-9 31836433 PMC6926471

